# Progression of scarring trachoma in Tanzanian children: A four-year cohort study

**DOI:** 10.1371/journal.pntd.0007638

**Published:** 2019-08-14

**Authors:** Athumani M. Ramadhani, Tamsyn Derrick, David Macleod, Patrick Massae, Elias Mafuru, Aiweda Malisa, Kelvin Mbuya, Chrissy h. Roberts, William Makupa, Tara Mtuy, Robin L. Bailey, David C. W. Mabey, Martin J. Holland, Matthew J. Burton

**Affiliations:** 1 London School of Hygiene and Tropical Medicine, London, United Kingdom; 2 Kilimanjaro Christian Medical Centre, Moshi, Tanzania; RTI International, UNITED REPUBLIC OF TANZANIA

## Abstract

**Background:**

Trachoma is a progressive blinding disease initiated by infection of the conjunctiva with *Chlamydia trachomatis*. Repeated infections are thought to cause chronic inflammation, which drives scarring, leading to in-turning of the eyelids. The relationship between *C*. *trachomatis*, clinical inflammation and scarring development in children is not fully understood due to a paucity of longitudinal studies with infection data at frequent follow-up.

**Methods and findings:**

This longitudinal cohort study took place in northern Tanzania. Children aged 6–10 years at baseline were eligible for inclusion. Participants were visited every three months for four years. Clinical signs and conjunctival swabs for *C*. *trachomatis* detection by qPCR were collected at each time-point. Conjunctival photographs from baseline and final time-points were graded and compared side-by-side to determine scarring incidence and progression.

Of the 666 children enrolled in the study, outcome data were obtained for 448. Scarring progression was detected in 103/448 (23%) children; 48 (11%) of which had incident scarring and 55 (12%) had progression of existing scarring. Scarring was strongly associated with increasing episodes of trachomatous papillary inflammation (TP). Weaker associations were found between episodes of *C*. *trachomatis* infection and follicular trachoma (TF) with scarring progression in unadjusted models, which were absent in multivariable analysis after adjusting for inflammation (multivariable results: *C*. *trachomatis* p = 0.44, TF p = 0.25, TP p = <0.0001, age p = 0.13, female sex p = 0.05). Individuals having TP at 30% or more of the time-points they were seen had an odds ratio of 7.5 (95%CI = 2.7–20.8) for scarring progression relative to individuals without any TP detected during the study period.

**Conclusions:**

These data suggest that the effect of infection on scarring progression is mediated through papillary inflammation, and that other factors contributing to the development of inflammation, in addition to *C*. *trachomatis* infection, may be important in driving conjunctival scarring progression in children. The addition of TP as a measure in trachoma control programs would provide an indication of the future risk of developing scarring sequelae.

## Author summary

Trachoma is the leading cause of preventable blindness worldwide and is targeted for elimination as a public health problem by 2020. The natural history of trachoma is not completely understood however. We conducted a four-year longitudinal study in a trachoma-endemic area of northern Tanzania with detailed follow up every three months. In the four-year study period, nearly one quarter of children developed progression of conjunctival scarring, despite three rounds of annual mass drug administration (MDA) for trachoma control. Disease progression was strongly associated with increasing proportion of episodes with conjunctival papillary inflammation (TP), and only weakly associated with *Chlamydia trachomatis* infection and *trachomatous inflammation–follicular* (TF). Analysis revealed that associations between infection and TF with scarring progression were mediated through TP, and that other factors causing individual differences in TP were also contributing to scarring progression. These data have significant implications for trachoma control. We hypothesise that in individuals who have previously experienced ocular *C*. *trachomatis* infection, TP is the primary driver of scarring progression. The addition of TP to trachoma surveillance programs would provide an indicator for active disease progression in the community and a more accurate guide to the need for future trichiasis interventions.

## Introduction

Sight loss from trachoma, the leading infectious cause of blindness, is the end result of an inflammatory-scarring process. Starting from early childhood, people growing-up in a trachoma endemic community may be repeatedly exposed to ocular challenge with *Chlamydia trachomatis*, the causative organism. This is thought to trigger inflammatory responses that lead to conjunctival scarring in some individuals[1]. As a result of conjunctival scarring the eyelids (entropion) and eyelashes (trichiasis) turn in, scratching the ocular surface and resulting in corneal opacification[1]. These complications of scarring usually develop during adulthood.

Trachoma control rests on the **SAFE** Strategy: **S**urgery for trichiasis, **A**ntibiotic treatment to treat *C*. *trachomatis* infection, **F**acial cleanliness and **E**nvironmental improvements to reduce transmission. Endemic countries and the international community have set the ambitious target of 2020 for the elimination of trachoma as a public health problem[2]. There is no specific treatment to halt the progression of scarring, beyond controlling the infection.

Around 3.2 million people are estimated to have trichiasis and 1.9 million of these are blind or have severe visual impairment[3]. Currently WHO estimates that 158 million people live in districts that require A, F and E interventions[4]. Nearly 90% of these people live in Sub-Saharan Africa.

Longitudinal data sets documenting the incidence or progression of conjunctival scarring trachoma are limited[1]. Such studies are complex and can take many years to complete. In this paper we report the clinical signs and infection results of children who were followed up every three months for four years. The aim of the study was to investigate the risk factors associated with scarring incidence and progression in Tanzanian children. We investigated the association between scarring progression and clinical signs of inflammation, *C*. *trachomatis* infection, age and sex, in order to strengthen the evidence base that supports trachoma control programs.

## Methods

### Ethical statement

This study was reviewed and approved by Ethics Committees of the Tanzania National Institute for Medical Research, Kilimanjaro Christian Medical University College and the London School of Hygiene & Tropical Medicine. It adhered to the tenets of the Declaration of Helsinki. The study was explained in detail in Kiswahili or Maasai; written informed consent from a parent or legal guardian was necessary for enrollment.

### Study design and population

We recruited a cohort of children from three neighboring villages in northern Tanzania. Two villages were in Siha district located in Kilimanjaro region and one was in Longido district located in Arusha region. They were assessed every three months for four years, totaling 17 time-points. The communities and recruitment have been previously described in detail[5]. These communities are predominantly comprised of Maasai people. Children aged between 6 and 10 years at baseline (February 2012), who were normally resident in the villages, were eligible for inclusion. This restricted age group was chosen as we anticipated that younger children may not have manifest incident / progressive conjunctival scarring during the four years of follow-up. A census was conducted and eligible children enrolled.

### Clinical assessment and sample collection

At each time-point all available children were examined by an experienced ophthalmic nurse. The eye was first anaesthetized with preservative-free proxymetacaine hydrochloride 0.5% eyedrops. The left upper eyelid was everted and tarsal conjunctiva examined (using x2.5 loupes and torch) for signs of trachoma and graded using the 1981 WHO ‘FPC’ detailed grading system[6]. This grading system corresponds to the WHO Simplified Trachoma Grading System: F2/F3 equates to *Trachomatous Inflammation-Follicular* (TF), and P3 to *Trachomatous Inflammation-Intense* (TI)[7]. “Clinically Active Trachoma” was defined as presence of TF and/or TI. We also consider that both P2 and P3 represent clinically significant papillary inflammation, and refer to this as “TP”[8]. High resolution photographs (Nikon D90 camera with 105mm Macro lens) were taken of the conjunctiva for independent grading.

Two conjunctival swab samples were collected (Dacron polyester, Puritan Medical Products Company, Maine) at each time-point. The first was placed in RNAlater (Thermo Fisher, UK) and the second was stored dry. Clinical swabs and air control swabs were collected and stored as described previously[5]. Samples were stored on ice in the field and were transferred to a -80°C freezer upon return to the laboratory later the same day.

### Trachoma control

Following approval from the Ministry of Health (MoH) and in collaboration with the district eye coordinators the SAFE strategy was implemented in study villages by the study field team. Education was provided regarding facial cleanliness and environmental improvements and free trichiasis surgery was offered. Azithromycin mass drug administration (MDA) was administered by the study team, according to WHO guidelines, in August 2012, August 2013 and August 2014. In mid-2015, one of the three villages from Longido district, which had a persistently elevated TF prevalence, received a further round of MDA. This was delivered by the local MoH team as part of the district-wide distribution. The other two villages, which are in the neighboring district (Siha), were not re-treated as the district-wide prevalence was below the treatment indication threshold, and these two villages had shown a good response to the three rounds of MDA.

### *Chlamydia trachomatis* detection

At the first time-point, DNA was extracted using the PowerSoil DNA Isolation Kit (Mo Bio Laboratories, USA) from swab samples stored in dry tubes and *C*. *trachomatis* was detected by droplet digital PCR, as previously described[5, 9]. At all subsequent time-points, DNA was extracted from samples stored in RNAlater using the Norgen RNA/DNA purification kit (Norgen Biotek) and *C*. *trachomatis* was detected by triplex quantitative PCR (qPCR) for chlamydial chromosomal (*omcB*) and plasmid (*pORF2*) genes and a human endogenous control gene (*RPP30*), as described previously[10]. Time-point 2 Norgen-extracted samples were tested by both detection methods and the kappa score for agreement was 0.84. Samples were tested in duplicate and were defined as *C*. *trachomatis* positive if *RPP30* and *pORF2* and/or *omcB* amplified in <40 cycles in one or both replicates.

### Analysis

We used photographic grading to determine whether there was either development of incident scarring in previously un-scarred conjunctiva, or increase in pre-existing scarring. Conjunctival photographs from baseline (time-point 1) were compared to the final time-point (time-point 17). For individuals not seen at time-point 1, the image from time-point 2 was used for their baseline. Similarly, if an individual was not seen at time-point 17, the image from time-point 16 was used as their final time-point. The images were assessed by an ophthalmologist experienced in using a detailed scarring grading system[11]. Baseline and final photographs were compared side-by-side to produce the main binary outcome variable of overall “scarring progression”, defined as evidence of either incident scarring or worsening of pre-existing scarring. For further sub-analyses, we subdivided individuals with “no scarring progression” into (1) no scarring at either baseline and final; (2) scarring unchanged between baseline and final. We subdivided individuals with “scarring progression” into (3) incident scarring (no scarring at baseline and new scarring at final); (4) increasing scarring (some scarring at baseline and more at final).

All field data were managed in Access (Microsoft). Data were merged and analyzed in STATA v14. The total number of time-points at which participants were seen varied due to absence or refusal. We excluded from the analysis individuals who were seen on fewer than four occasions or did not have outcome data (time-point 16 or 17 assessments). A proportion variable was generated for each of TF, TP and *C*. *trachomatis* infection: number of time-points with each factor as a proportion of the total number of time-points that individual was seen. Proportions were subsequently categorized.

Separate mixed effects logistic regression models were used to determine the association between (1) TP, (2) TF, and (3) *C*. *trachomatis* infection with sex and baseline age, using data from all time-points in the longitudinal dataset. Mixed effects regression was also performed to assess the relationship between (1) TF and *C*. *trachomatis* infection, and (2) TP and *C*. *trachomatis*, again using data from all time-points in the longitudinal dataset (adjusting for age at baseline and sex). These analyses were limited to the 448 individuals with outcome data.

To identify risk factors for scarring progression, analysis was initially performed using logistic regression to assess the association between categorized proportions of TF, TP or *C*. *trachomatis* infection and overall scarring progression (either incident scarring in those without scarring at baseline or progression of pre-existing scarring). Each of these were initially included as exposures separately in a logistic regression using scarring progression as the outcome variable and adjusting for age at baseline and sex. Following this, all three were included in a final multivariable model (adjusting for baseline age and sex), and likelihood ratio tests were performed between models including versus excluding each exposure to determine its overall *P* value. The analyses were subsequently repeated to identify risk factors for incident scarring and progression of pre-existing scarring separately. In the first set of univariable and multivariable analyses (using the same exposures as above) the analysis was restricted to individuals without scarring at baseline (incident scarring versus no scarring). In the second set, analysis was restricted to individuals with scarring at baseline (progression of existing scarring versus no progression of existing scarring).

Chlamydial load was calculated by extrapolating from a standard curve. *OmcB* in copies/μl was log10 transformed to normalize the distribution. In the longitudinal dataset random effects linear regressions were performed to look for associations between a) chlamydial load and scarring progression (adjusting for MDA-period, age at baseline and sex), and b) chlamydial load and age at time-point (in years) in *C*. *trachomatis* positive individuals (adjusting for sex and MDA-period). Age at time-point was split into four groups; <7.5 years, ≥7.5 - <10 years, ≥10 - <12.5 years, and ≥12.5 years. A random effects linear regression was then performed to assess for association between chlamydial load, age group and progression, including an interaction term between age group and progression in order to determine whether the association between chlamydial load and progression was modified by age. MDA-period (pre-MDA, post first MDA, post second MDA, post third MDA) was included in the model to adjust for confounding.

## Results

### Study participants

**Fig 1:**
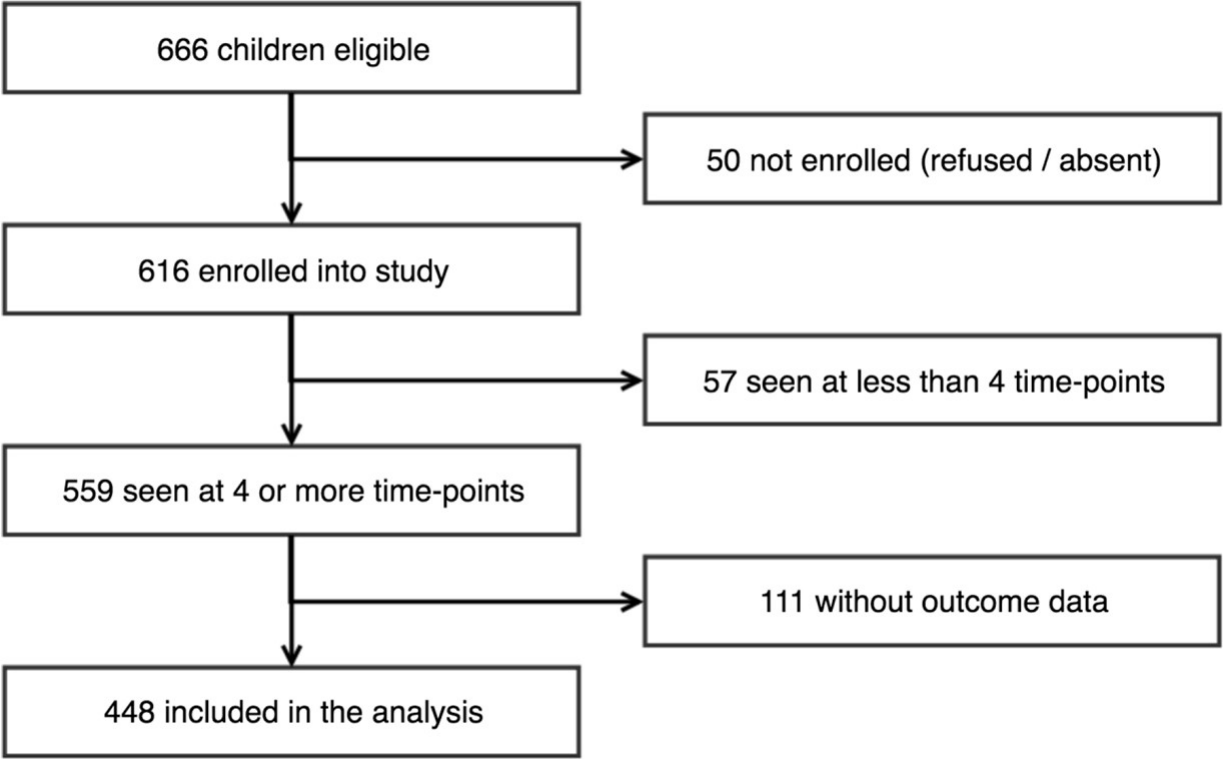
Flow chart for eligible study participants. The chart shows the number of individuals enrolled, excluded and included in the analysis of scarring progression.

The participant flow is shown in Fig 1. There were 666 potentially eligible children and 616 enrolled. Fifty either refused or were absent. We excluded 57 who were examined on less than four occasions, and 111 without scarring progression outcome data (no time-point 16 or 17 assessment). This left 448 in this analysis, who were seen at a median of 15 time-points (1^st^– 3^rd^ quartiles = 13–16, S1 Fig).

The demographic characteristics of the entire cohort were described in the baseline report[5]. Of the 448 children included in this analysis, 242 (54.0%) were female, mean age at baseline was 6.8 years, and 438 (97.8%) were Maasai. Of the 218 children not included, 92 (42.2%) were female (OR = 1.61, 95%CI = 1.16–2.23, p = 0.004), mean age at baseline was 7.4 years (OR = 0.86, 95% CI = 0.79–0.93, p<0.0002) and 214 (98.2%) were Maasai. Younger children and females were therefore more likely to be included in this study analysis.

Antibiotic coverage of the 448 children included in scarring progression analysis was 355 (79.2%) in 2012, 374 (83.5%) in 2013 and 338 (75.4%) in 2014. The estimated community-wide MDA coverage in 2012, 2013 and 2014 were 68.7%, 42.9% and 72.9%, respectively.

### Clinical disease

**Fig 2:**
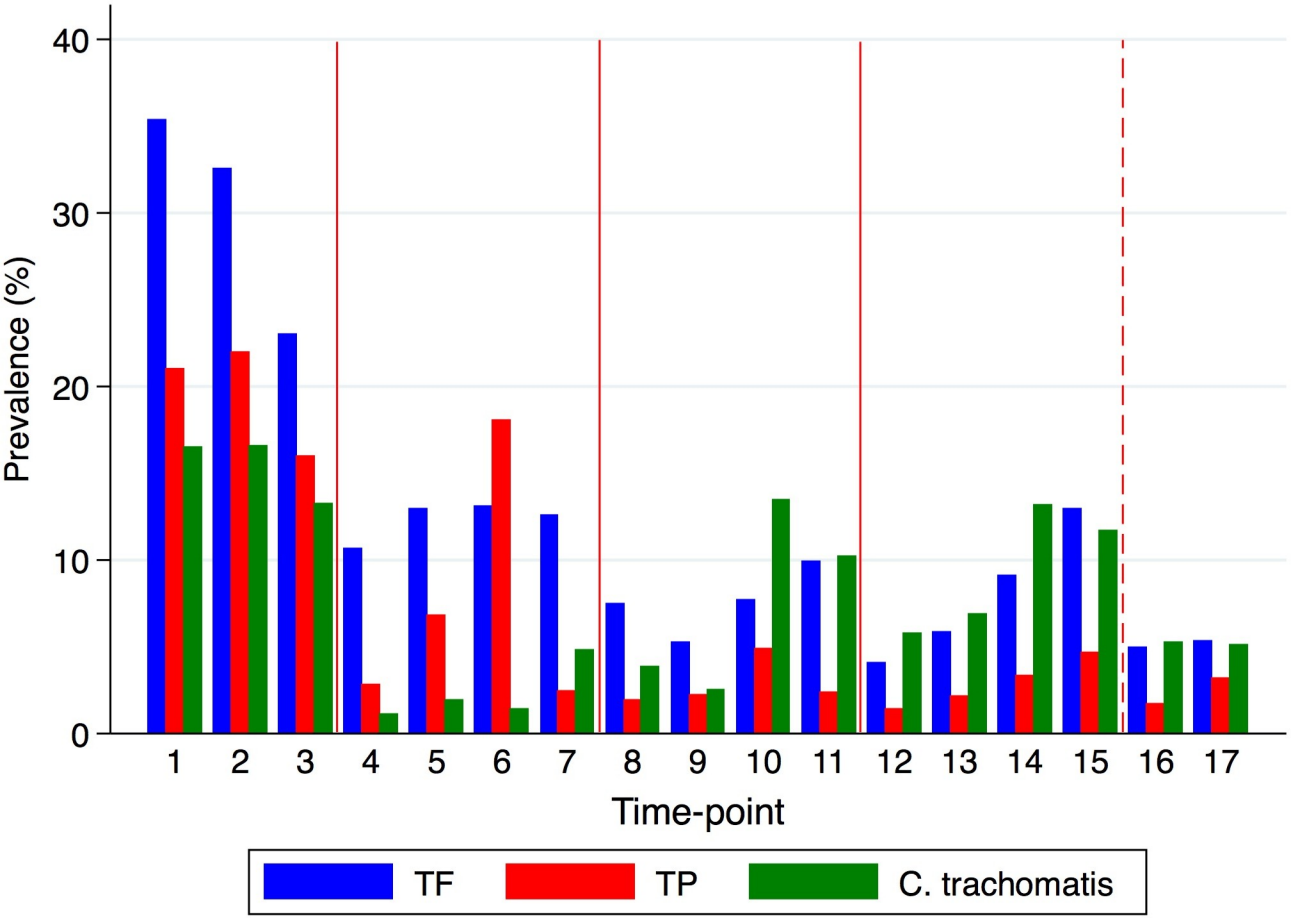
The prevalence of *C*. *trachomatis* infection and clinical signs at each time-point. Data are shown for the 448 individuals with outcome data. There were three-month intervals between time-points. Red vertical lines indicate annual MDA treatment given to all three study villages. The red dashed vertical line indicates treatment given to one village with residual disease.

Of the 448 participants, 240 (53.6%) had TF (F2/F3), 185 (41.3%) had TP (P2/P3)), and 248 (55.4%) had clinically active trachoma (F2/3 and/or P3) at one or more time-point. The prevalence of TF and TP is shown for each time-point in Fig 2.

There was a significant reduction in inflammatory disease following MDA, although TP prevalence was particularly high at time-point 6. Examination of inflammation and infection prevalence by village revealed that this peak in TP was found in only two of the three villages (“A” and “C”), and did not appear to correlate with infection (S2 Fig), indicating that it may have been driven by non-chlamydial infection.

The number of individuals with categorized proportions of time-points (none, <10%, 10–19%, 20–29%, 30%+) that they were found to have (1) TF, (2) TP and (3) *C*. *trachomatis* infection is shown in Table 1. At baseline, 93 (20.8%) had some degree of conjunctival scarring.

**Table 1:** Number of individuals with *C*. *trachomatis* infection, TF and TP, categorized by proportion of time-points. *C*. *trachomatis* infection, TF and TP were detected as a percentage of the total number of time-points that individual was seen.

Proportion of time-points	*C*. *trachomatis*		TF		TP	
	n	(%)	n	(%)	n	(%)
None	229	(51.1%)	208	(46.4%)	263	(58.7%)
<10%	78	(17.5%)	52	(11.6%)	71	(15.8%)
10–19%	79	(17.6%)	71	(15.9%)	48	(10.7%)
20–29%	43	(9.6%)	56	(12.5%)	41	(9.2%)
30% +	19	(4.2%)	61	(13.6%)	25	(5.6%)

The odds of TF were estimated to be higher in females (OR = 1.49, 95%CI = 1.05–2.11, P = 0.025) and lower with each additional year of age (OR = 0.65, 95%CI = 0.59–0.71, p<0.0001) in the longitudinal dataset. The odds of TP were also estimated to reduce with age (OR = 0.79, 95%CI = 0.71–0.88, p<0.0001) but the evidence of an association with sex was much weaker (OR = 1.39, 95%CI = 0.92–2.09, p = 0.119). There was little difference between children included and those excluded in the analysis in terms of sex and baseline TF/TP/infection although those excluded tended to be slightly older on average than those included (mean baseline age 7.4 vs 6.8, p<0.001).

### *C*. *trachomatis* infection

*C*. *trachomatis* was detected in 219/448 (48.9%) at one or more time-points. The prevalence of infection is shown for each time-point in Fig 2. The proportion of time-points that each individual had infection is shown in Table 1. The median proportion of time-points infected among the 219 individuals who had *C*. *trachomatis* detected on at least one occasion was 12.5%, which was equivalent to ~2 time-points if someone had been seen on all 17 visits.

Infection prevalence declined following each round of MDA, however at time point 10 (9 months after second MDA) it had increased and at time-point 14 (9 months after third MDA) infection prevalence had returned to pre-MDA levels (10–15%). Infection prevalence dropped again by time-points 16 and 17. Further examination of infection and clinical sign prevalence in each of the three villages showed that the majority of infection and TF at later time-points were found in only village “C” (S2 Fig). Village “C” is located in a different administrative district, which (unlike the other two villages) was eligible for and received MoH administered MDA treatment in July-2015. This was subsequently followed by a further drop in infection, TF and TP prevalence in village “C” at time-points 16 and 17 (S2 Fig).

In a mixed effects logistic regression of infection at any time-point, female sex (OR = 1.7, 95%CI = 1.25–2.34, p = 0.001) and younger baseline age (OR = 0.82, 95%CI = 0.75–0.99, p<0.0001) were significantly associated with *C*. *trachomatis* infection.

### Clinical disease and infection

Overall, there was a strong association between *C*. *trachomatis* infection and TF (OR = 11.6, 95%CI = 8.9–15.0, P<0.0001) and TP (OR = 9.6, 95%CI = 7.1–12.8, P<0.0001) in the longitudinal dataset (adjusted for baseline age and sex). The odds ratios for TP and TF as predictors of *C*. *trachomatis* infection (adjusted for TP/TF, age at baseline and sex) at each time-point were generally similar to or slightly higher after the initiation of MDA, however confidence intervals were much wider (S3 Fig).

### Scarring progression

Overall, scarring progression was observed in 103/448 (23.0%) participants (Table 2). There were 307 (68.5%) who had no scarring; 38 (8.5%) with unchanged scarring; 48 (10.7%) with incident scarring; and 55 (12.3%) with increasing scarring.

**Table 2:** Scarring progression category by presence of *C*. *trachomatis* infection and clinical features. Both infection and clinical features were detected at one or more time-points.

		Overall scarring		Scarring subgroups			
Clinical phenotype	Total	Progression	No progression	No scarring	Unchanged scarring	Incident scarring	Increasing scarring
Total	448	103 (23%)	345 (77%)	291 (65%)	54 (12%)	48 (10.7%)	55 (12.3%)
No infection/TP/TF	122	17 (13.9%)	105 (86.1%)	95 (77.9%)	10 (8.2%)	11 (9%)	6 (4.9%)
Any TF	240	67 (27.9%)	173 (72.1%)	136 (56.7%)	37 (15.4%)	31 (12.9%)	36 (15%)
Any TP	185	67 (36.2%)	118 (63.8%)	89 (48.1%)	29 (15.7%)	28 (15.1%)	39 (21.1%)
Any *C*. *trachomatis*	219	55 (25.1%)	164 (74.9%)	137 (62.6%)	27 (12.3%)	29 (13.2%)	26 (11.9%)

The relationships between scarring progression and proportion of time-points when *C*. *trachomatis* was detected or signs of inflammation (TF or TP) were seen, adjusting only for age at baseline and sex, are shown in Table 3. In these models strong evidence was found of an association between progression and both TP and TF, but the association between infection and progression was weaker. There was also evidence of a greater risk of progression in females compared to males.

**Table 3:** Univariable logistic regression models of the associations between overall scarring progression and *C*. *trachomatis* infection and clinical features. *C*. *trachomatis* infection, TF and TP were categorized by proportions of time-points present, and were adjusted only for age at baseline and sex. The overall *P* value for each model is shown in line with the variable name.

	n/N	(%)	OR	95% CI	P value
***C*. *trachomatis***					0.041
None	48/229	(20.9%)			
<10%	12/78	(15.4%)	0.64	0.3–1.3	
10–19%	21/79	(26.6%)	1.29	0.7–2.4	
20–29%	15/43	(34.9%)	1.91	0.9–3.9	
30% +	7/19	(36.8%)	2.20	0.8–6.0	
**TF**					0.0004
None	36/208	(17.3%)			
<10%	8/52	(15.4%)	0.93	0.4–2.2	
10–19%	18/71	(25.4%)	1.96	1.0–3.9	
20–29%	15/56	(26.8%)	1.92	0.9–4.0	
30% +	26/61	(42.6%)	4.41	2.2–8.8	
**TP**					<0.0001
None	36/263	(13.7%)			
<10%	17/71	(23.9%)	2.14	1.1–4.2	
10–19%	15/48	(31.3%)	3.08	1.5–6.4	
20–29%	21/41	(51.2%)	7.26	3.5–15.0	
30% +	14/25	(56.0%)	8.41	3.5–20.2	
**Age at baseline**			1.01	0.9–1.1	0.853
**Sex** Male Female	38/206 65/242	(18.5%) (26.9%)	1.62	1.0–2.6	0.036

In a multivariable model (Table 4) for scarring progression (retaining infection, TF, TP, age at baseline and sex), the strong relationship between increasing proportion of time-points with TP and scarring progression remained. Female sex was marginally associated. There was no association with either TF or infection, suggesting that the associations between TF and infection with scarring were mediated through TP.

**Table 4:** Multivariable logistic regression model for the association between overall scarring progression and *C*. *trachomatis* infection and clinical features. Categorized proportions of time-points with *C*. *trachomatis* infection, TF and TP were included, adjusting for age at baseline and sex. The overall P value for each variable is shown in line with the variable name, derived from a likelihood ratio test of the model including versus excluding that variable.

	OR	95% CI	P value
***C*. *trachomatis***			0.4396
None			
<10%	0.49	0.2–1.1	
10–19%	0.90	0.5–1.8	
20–29%	0.80	0.3–1.9	
30% +	0.72	0.2–2.5	
**TF**			0.2535
None			
<10%	0.85	0.3–2.1	
10–19%	1.07	0.5–2.3	
20–29%	0.84	0.3–2.0	
30% +	2.09	0.9–5.0	
**TP**			<0.0001
None			
<10%	2.19	1.1–4.4	
10–19%	2.94	1.3–6.7	
20–29%	6.67	3.0–14.9	
30% +	7.48	2.7–20.8	
**Age at baseline**	1.11	1.0–1.3	0.128
**Sex (Female)**	1.65	1.0–2.7	0.051

The analysis was repeated, restricted to individuals with (a) no scarring at baseline, and (b) some scarring at baseline, in order to differentiate between factors associated with incident scarring and progression of pre-existing scarring, respectively. In the unadjusted models there was evidence for associations between episodes of *C*. *trachomatis* infection, TF and TP and incident scarring (S1 Table). In the multivariable model however, only TP was significantly associated with incident scarring, again suggesting that the effect of infection and TF was mediated through TP (S2 Table). Neither infection, TF nor TP were significantly associated with progression of pre-existing scarring in either the unadjusted or adjusted models. There seemed to be a trend for increasing risk of progressive scarring with increasing episodes of TP, however the evidence for this effect was weak, it should be noted that the sample size for these sub-analyses was small. These data from children with pre-existing scarring did not demonstrate that additional episodes of *C*. *trachomatis* infection were associated with further progression of scarring. Female sex was associated with an increase in pre-existing scarring but not with incident scarring. There were no associations with age.

### *C*. *trachomatis* infection load

**Fig 3:**
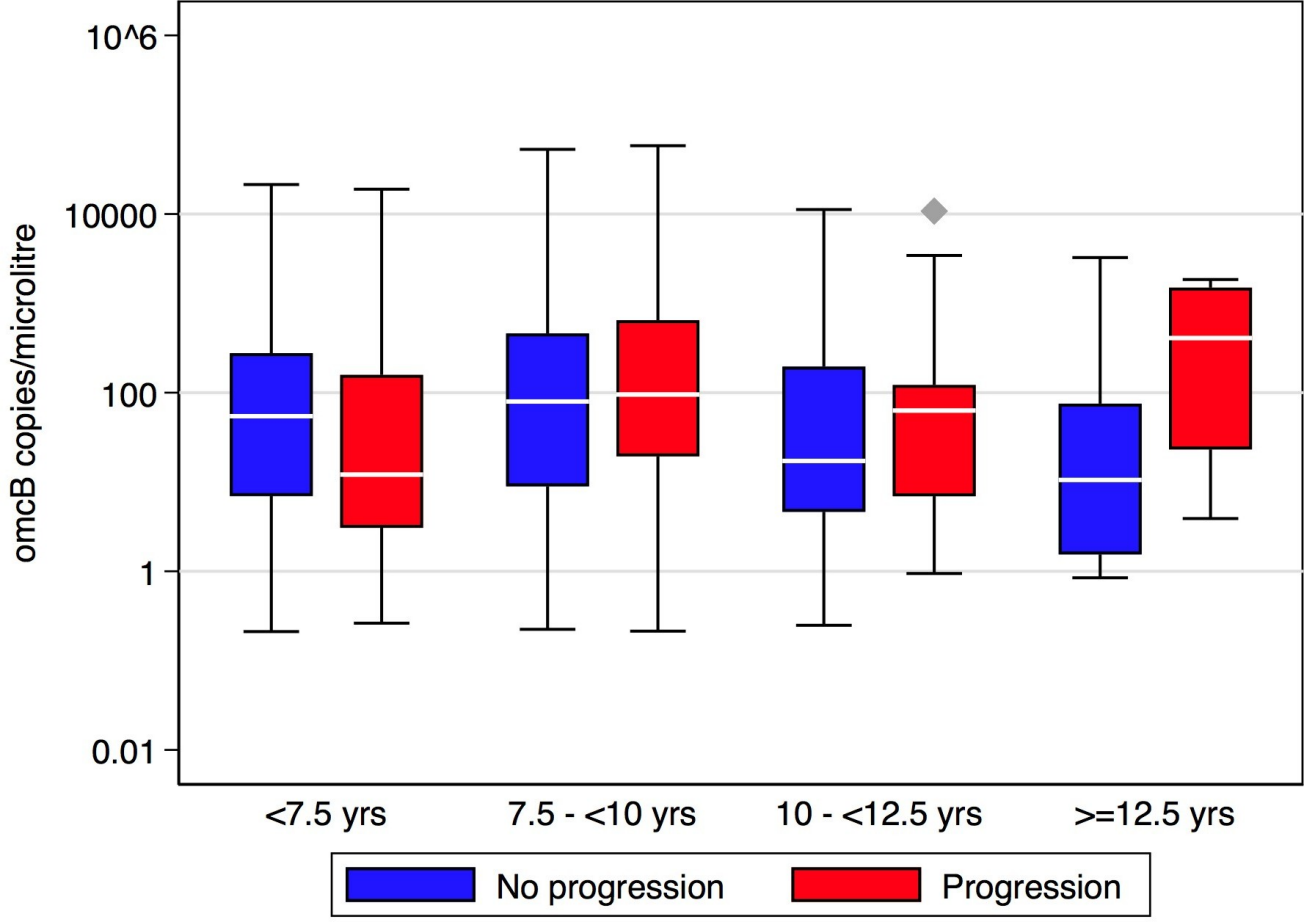
Distribution of *omcB* load in scarring progressors and non-progressors, split by age group at time-point.

Bacterial load in *C*. *trachomatis* positive individuals was equivalent between people with and without scarring progression, using data from all time-points (adjusting for age at baseline, sex and pre/post-MDA period) (OR = 1.1, 95% CI = 0.86–1.42, p = 0.45). There was evidence of an association between age at time-point (in years) and infection load among *C*. *trachomatis* positive individuals, with lower loads in older individuals (OR = 0.91, 95%CI = 0.86–0.97, p = 0.004), adjusting for sex and MDA period. Bacterial load in *C*. *trachomatis* positive progressors and non-progressors was plotted across different age groups (derived from age in years at that time-point) to determine whether the association between scarring progression and load varied by age. In the oldest age group, progressors had a slightly higher infection load relative to non-progressors, Fig 3, which was supported by evidence for an interaction between age group and progression in their association with bacterial load (p = 0.012). The model including the interaction explained the data better than the model without the interaction (p = 0.016).

## Discussion

The development of trachomatous scarring is probably the result of a complex interaction between *C*. *trachomatis* infection, variation in the host immune response and possibly other pro-inflammatory stimuli. However, long-term data exploring the relative contributions of these factors to the development of scarring in children are limited.

In this cohort of Tanzanian children aged 6–10 years old at baseline we found that 23% had trachomatous scarring progression over the course of four years. Roughly half of this was new scarring whilst the other half involved progression of pre-existing scarring. Scarring progression was strongly associated with increasing frequency of conjunctival papillary inflammation (TP). This suggests that controlling TP could potentially limit progressive scarring trachoma.

We found only weak evidence of an association between *C*. *trachomatis* infection and scarring progression. Increasing proportions of infection episodes were associated with incident scarring, however, multivariable analysis showed that this effect was mediated through TP. These data suggest that other factors, in addition to *C*. *trachomatis* infection, are important determinants of the development of TP and progression of scarring. The data also indicate that the clinical sign of TF has no association with scarring progression after adjusting for TP, suggesting that TF is not a direct cause of, nor the best prognostic marker for scarring progression. For this purpose, TP prevalence might be a more informative clinical marker, and control programs could consider using TF in combination with TP prevalence to predict future risk of scarring.

Our finding that increasing frequency of papillary inflammation is strongly associated with scarring progression has consistently been reported by other studies[1], however the relationship between chlamydial infection and scarring progression is less definitive.

The first longitudinal study to try to investigate scarring progression was conducted by Dawson et al in Tunisia, starting in the late 1960’s. They examined a group of children and younger adults (n = 213) on two occasions about 14 years apart; no tests for *C*. *trachomatis* infection were performed. TI (P3) was the strongest risk factor for developing severe scarring (RR = 18), whilst TF had a weaker association (RR = 2.8)[12]. Interestingly, there also appeared to be increased scarring risk associated with moderate papillary inflammation (P2).

West et al investigated the relationship between “constant severe inflammatory trachoma” (TI diagnosed at more than half of the examinations) in a group of children (n = 190) examined on four occasions during the baseline year and once again at 7 years[13]. TI was associated with increased risk of developing scarring by 7 years. TF alone was not associated with development of scarring.

Only one study, by Wolle et al, has previously examined the relationship between *C*. *trachomatis* infection and the subsequent development of scarring in children[14]. They reported a five-year cohort of Tanzanian children (n = 189) that were examined on five occasions during the first 18 months and once again at 5 years. They found that incident scarring over the five years was associated with constant inflammation and/or constant *C*. *trachomatis* infection during the first 18 months (OR 5.74, 95%CI 2.39–13.8). However, the effects of infection and inflammation were not modelled independently of each other, possibly due to sample size limitations, therefore, the independent contribution from infection remained unclear.

Burton et al found a strong association between progression of pre-existing scarring in adults and increasing episodes of papillary inflammation (P2/P3) in Ethiopia (n = 585; OR 5.93, 95%CI 3.31–10.6, p<0.0001) and Tanzania (n = 577; OR 5.76, 95%CI 2.60–12.7, p<0.0001)[8]. The study examined and sampled participants on a 6-monthly basis for two years. Episodes of *C*. *trachomatis* infection were very infrequent, and they were not associated with disease progression.

Our finding that *C*. *trachomatis* infection was marginally associated with incident scarring (before adjustment) but not with progressive scarring is consistent with chlamydial infection being important for initiating the scarring disease process. However, once scarring is established, other factors driving TP are perhaps increasingly important for scarring progression. However, an alternative but perhaps less likely explanation for this finding might be that people who are more innately predisposed to developing scarring clear infection episodes rapidly (which are therefore not readily detected), but also experience more severe and prolonged inflammation (TP) in the aftermath[15, 16]. Our analysis of progression of pre-existing scarring was also limited by a smaller sample size.

Conjunctival inflammation may be associated with other bacterial or viral infections, or with allergic conjunctivitis[17, 18]. Several cross-sectional studies have found associations between non-chlamydial ocular bacterial infections and active trachoma (TF/TP), conjunctival scarring, trichiasis/recurrent trichiasis and corneal scarring[11, 19–24]. A recent longitudinal study in 452 Tanzanian adults found that ocular commensal and pathogenic non-chlamydial bacterial infections were more common in scarring progressors relative to non-progressors and that, after adjusting for other factors, ocular infections were marginally associated with scarring progression at two years[25].

*C*. *trachomatis* infection might damage the barrier function or homeostasis of the conjunctival epithelium, such that external stimuli cause inflammation where they would not have done otherwise. The expression of mucins has consistently been found to be dysregulated in active and scarring trachoma, supporting this hypothesis[5, 26]. Long-term exposure to cooking smoke has also been linked to conjunctival inflammation[27], particularly affecting women, however a Tanzanian longitudinal study did not find any association between exposure to cooking fires and incident scarring[28]. Use of traditional medicines might also have a role, as could differences in diet or coinfections that lead to variation in host immune responses.

Genetic or epigenetic factors might also contribute to differences in host inflammatory responses. Several studies have reported associations between genetic differences and risk of trachoma, including one genome-wide association study[29–35]. Further detailed investigations of ocular microbial infections and host genetics are required to establish their roles in this disease.

There was a marginal association between female sex and overall scarring progression. Further analysis revealed that female sex was associated with progression of pre-existing scarring but not with incident scarring (S1 Table). Female sex was also associated with *C*. *trachomatis* infection, TF and only very marginally with TP. These findings are difficult to reconcile, as there were associations between infection and incident scarring, and between infection and female sex. Whereas, there was no association between infection and progressive scarring and only a marginal association between TP and female sex. Our findings also contrast those of previous longitudinal studies in which female sex was associated with scarring incidence but not progression[1]. The numbers in our analysis of pre-existing scarring were relatively small, therefore sample size limitations might explain these findings. Nevertheless, overall females were at greater risk of overall scarring progression, after adjustment for *C*. *trachomatis* infection and TP, suggesting that this effect was mediated by another mechanism.

We found no association between age and scarring progression, which may be due to the limited four year age range of our study participants. Age was strongly associated with *C*. *trachomatis* infection, TF and TP, all of which were more common in younger participants, perhaps suggesting that some level of acquired protective immunity develops. There was some evidence that in the oldest age group, scarring progressors had a higher chlamydial load relative to non-progressors, perhaps suggesting that their immunological control of the infection was less effective, although this evidence was very weak.

A strong association was seen between infection and TF or TP. In contrast to previous reports the association did not diminish after MDA[36], however *C*. *trachomatis* infection was still relatively common and the effect estimates had very wide confidence intervals. Infection prevalence recovered after MDA to near pre-MDA levels, whereas TP and TF prevalence remained low post-MDA. Most of the residual disease and infection was concentrated in village “C”, which lies within a district with historically higher levels of endemic trachoma. A study from central Tanzania has shown a significant reduction of both *C*. *trachomatis* infection and TF after each of three annual rounds of MDA in children aged 1 year and above, where MDA coverage was >80%[37]. The overall TF prevalence was reduced from 27.3% at baseline to 9.4% one year after the third MDA round, and infection prevalence was reduced from >20% in 1-9-year olds to <10% in the fourth year. The resurgence of infection prevalence during our study may have been due to a combination of insufficient MDA coverage and interaction with untreated neighboring communities. Community members are mainly pastoralists and often away from their homes during the day. These results highlight the need for high MDA coverage to effectively bring *C*. *trachomatis* prevalence under control. However, the sustained reduction in TP prevalence following MDA may be promising in terms of reducing scarring risk.

This study has several limitations. Only the left eye was examined and sampled throughout the longitudinal study as it was not feasible to process samples from both eyes at all time-points. There were changes in the detection method for *C*. *trachomatis* between baseline and all other time-points, which could have introduced some variability, however the agreement between methods was good and due to the large sample size and high number of time-points the interpretation of the results is not expected to be affected. As with any longitudinal study there was some loss to follow-up. In this study we found that the children for whom we were unable to collect outcome data were slightly older and more likely to be male. Although this moderate difference between those seen and not seen could potentially introduce some bias, the cohort size remained substantial to the end, and despite the loss to follow-up we were able draw clear conclusions. It is possible that MDA treatment could have had a beneficial effect against scarring progression through the known anti-inflammatory properties of azithromycin, however further investigation of this was outside the scope of the current study. The three-monthly spacing of observations are too far apart to be able to estimate the duration of disease and infection episodes. The study was designed to investigate individual level risk of progression in scarring and was of sufficient size to demonstrate this. However, it was not designed to provide community level estimates of the risk of progression, which would require a much larger number of communities and many more to children.

### Conclusion

Progressive scarring trachoma was strongly associated with papillary inflammation in this longitudinal study. *C*. *trachomatis* infection was no longer associated with scarring progression after adjustment for TP, suggesting that the effect of infection is mediated through TP, and that other factors contributing to TP in addition to *C*. *trachomatis* infection are important determinants of disease progression. Further research is required to understand what these factors are; they might include other ocular or non-ocular infections, genetic variation in host immune responses or environmental factors. Females were at greater risk of *C*. *trachomatis* infection, clinical inflammation and scarring progression. The addition of TP as an indicator for trachoma control programs might provide a more accurate marker for the risk of disease progression and of the need for future trichiasis interventions, which are likely to be needed for many years to come in this community.

## Supporting information

S1 Checklist STROBE checklist. (DOC)

S1 Fig Histogram showing the number of time-points at which participants were seen. Data are shown for the 448 participants with outcome data.
(TIF)

S2 Fig The prevalence of *C*. *trachomatis* infection and clinical signs at each time-point. Data are shown for the 448 individuals with outcome data, split by village. Red vertical lines indicate MDA treatment. The red dashed vertical line in village C indicates treatment given with residual disease
(TIF)

S3 Fig The association between *C*. *trachomatis* infection and clinical signs at each time-point. TF is shown in blue and TP in red. Odds ratios with 95% confidence intervals are plotted. The grey vertical lines indicate MDA treatment given to all three study villages. The grey dashed vertical line indicates treatment given to one village with residual disease. The OR for TP at time-point 5 is missing as there was insufficient data to generate a result.
(TIF)

S1 Table Univariable logistic regression models for scarring progression. These include; a) incident scarring and b) increase in pre-existing scarring, in individuals with (a) no scarring at baseline or (b) some scarring at baseline. Univariate associations between scarring and infection, TF and TP were adjusted for age at baseline and sex.
(DOCX)

S2 Table Multivariable logistic regression models for scarring progression. These include; a) incident scarring and b) increase in pre-existing scarring, in individuals with (a) no scarring at baseline or (b) some scarring at baseline.
(DOCX)
