# Supplementary figures and images for: Progression of scarring trachoma in Tanzanian children: A four-year cohort study
Source: PLoS Negl Trop Dis. 2019 Aug 14;13(8):e0007638. doi: 10.1371/journal.pntd.0007638 (PMC6709924; doi:10.1371/journal.pntd.0007638)

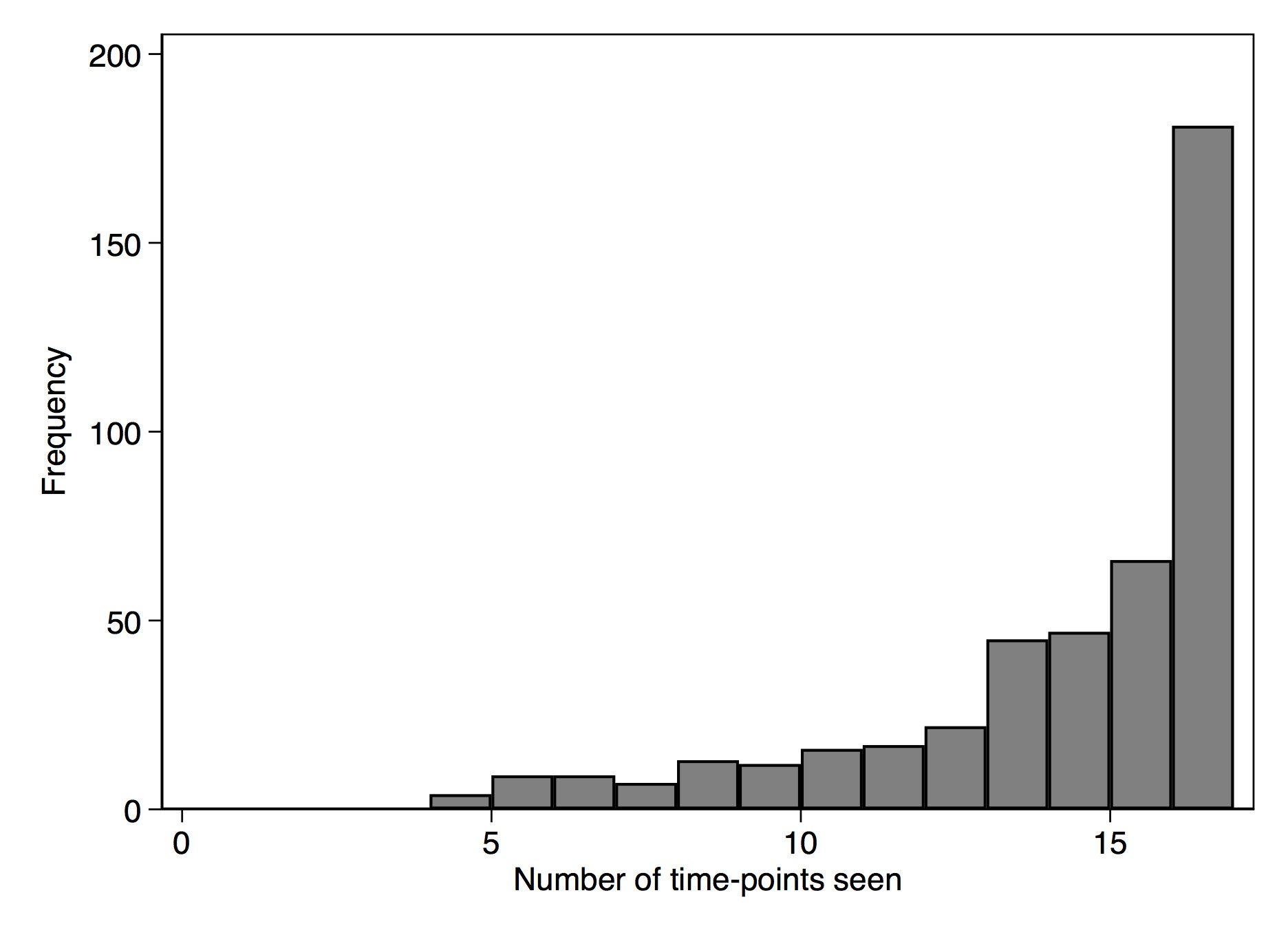

Supplement: S1 Fig — Data are shown for the 448 participants with outcome data. (TIF) [file pntd.0007638.s002.tif]

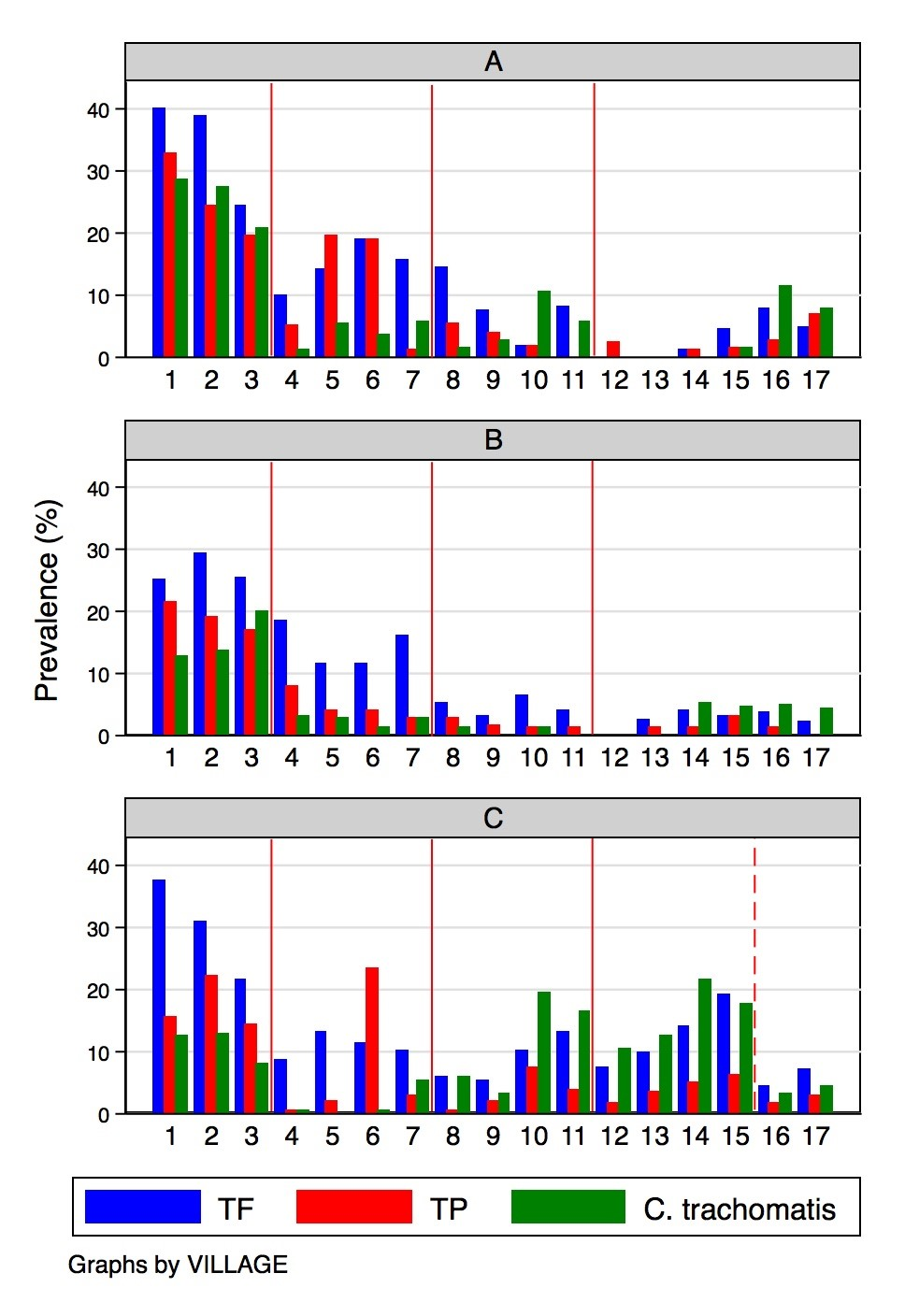

Supplement: S2 Fig — Data are shown for the 448 individuals with outcome data, split by village. Red vertical lines indicate MDA treatment. The red dashed vertical line in village C indicates treatment given with residual disease (TIF) [file pntd.0007638.s003.tif]

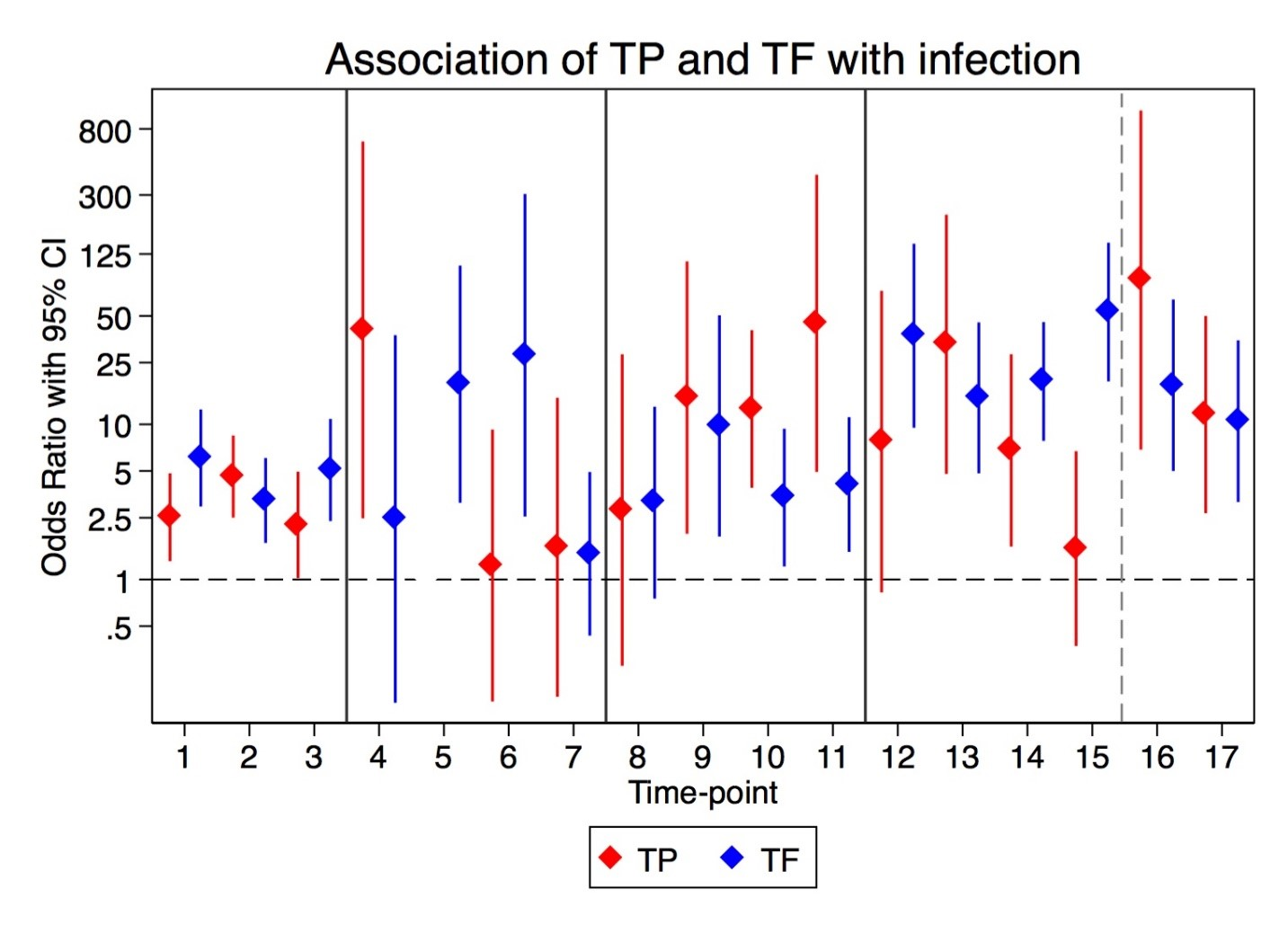

Supplement: S3 Fig — TF is shown in blue and TP in red. Odds ratios with 95% confidence intervals are plotted. The grey vertical lines indicate MDA treatment given to all three study villages. The grey dashed vertical line indicates treatment given to one village with residual disease. The OR for TP at time-point 5 is missing as there was insufficient data to generate a result. (TIF) [file pntd.0007638.s004.tif]
