# Supplementary material for: Progression of scarring trachoma in Tanzanian children: A four-year cohort study
Source: PLoS Negl Trop Dis. 2019 Aug 14;13(8):e0007638. doi: 10.1371/journal.pntd.0007638 (PMC6709924; doi:10.1371/journal.pntd.0007638)
Supplement: S1 Table — These include; a) incident scarring and b) increase in pre-existing scarring, in individuals with (a) no scarring at baseline or (b) some scarring at baseline. Univariate associations between scarring and infection, TF and TP were adjusted for age at baseline and sex. (DOCX) [file pntd.0007638.s005.docx]

**S1 Table. Univariate logistic regression models for scarring progression.** That includes; a) incident scarring and b) increase in pre-existing scarring, in individuals with (a) no scarring at baseline or (b) some scarring at baseline. Univariate associations between scarring and infection, TF and TP were adjusted for age at baseline and sex

| **A) INCIDENT SCARRING** | **n/N** | **%** | **OR** | **95% CI** | **P value** |
| --- | --- | --- | --- | --- | --- |
|  | 48/339 | 14.16 |  |  |  |
| ***C. trachomatis*** |  |  |  |  | 0.0307 |
| None | 19/173 | (11.0%) |  |  |  |
| <10% | 5/67 | (7.5%) | 0.68 | 0.24 - 1.91 |  |
| 10-19% | 10/53 | (18.9%) | 1.98 | 0.84 - 4.64 |  |
| 20-29% | 9/32 | (28.1%) | 3.40 | 1.33 - 8.68 |  |
| 30% + | 5/14 | (35.7%) | 4.75 | 1.42 - 15.90 |  |
| **TF** |  |  |  |  | 0.0013 |
| None | 17/172 | (9.9%) |  |  |  |
| <10% | 2/37 | (5.4%) | 0.62 | 0.13 - 2.86 |  |
| 10-19% | 6/47 | (12.8%) | 1.63 | 0.58 - 4.58 |  |
| 20-29% | 8/43 | (18.6%) | 2.54 | 0.96 - 6.71 |  |
| 30% + | 15/40 | (37.5%) | 7.35 | 2.91 - 18.54 |  |
| **TP** |  |  |  |  | <0.0000 |
| None | 20/222 | (9.0%) |  |  |  |
| <10% | 4/49 | (8.2%) | 0.99 | 0.32 - 3.09 |  |
| 10-19% | 5/29 | (17.2%) | 2.40 | 0.80 - 7.19 |  |
| 20-29% | 15/28 | (53.6%) | 13.42 | 5.34 - 33.73 |  |
| 30% + | 4/11 | (36.4%) | 6.03 | 1.61 - 22.63 |  |
| **Age at baseline** |  |  | 0.99 | 0.84 - 1.15 | 0.859 |
| **Sex** |  |  |  |  |  |
| Male | 24/168 | (14.3%) |  |  |  |
| Female | 24/171 | (14.0%) | 0.98 | 0.53 - 1.80 | 0.947 |

| **B) PROGRESSIVE SCARRING** | **n/N** | **%** | **OR** | **95% CI** | **P value** |
| --- | --- | --- | --- | --- | --- |
|  | 55/109 | (50.5%) |  |  |  |
| ***C. trachomatis*** |  |  |  |  | 0.3973 |
| None | 29/56 | (51.8%) |  |  |  |
| <10% | 7/11 | (63.6%) | 0.93 | 0.21 - 4.18 |  |
| 10-19% | 11/26 | (42.3%) | 0.51 | 0.18 - 1.46 |  |
| 20-29% | 6/11 | (54.6%) | 0.80 | 0.20 - 3.18 |  |
| 30% + | 2/5 | (40.0%) | 0.62 | 0.09 - 4.36 |  |
| **TF** |  |  |  |  | 0.5382 |
| None | 19/36 | (52. 8%) |  |  |  |
| <10% | 6/15 | (40.0%) | 0.68 | 0.19 - 2.40 |  |
| 10-19% | 12/24 | (50.0%) | 1.01 | 0.33 - 3.09 |  |
| 20-29% | 7/13 | (53.9%) | 1.19 | 0.31 - 4.60 |  |
| 30% + | 11/21 | (52.4%) | 0.87 | 0.26 - 2.91 |  |
| **TP** |  |  |  |  | 0.1783 |
| None | 16/41 | (39.0%) |  |  |  |
| <10% | 13/22 | (59.1%) | 2.17 | 0.73 - 6.46 |  |
| 10-19% | 10/19 | (52.6%) | 1.79 | 0.57 - 5.63 |  |
| 20-29% | 6/13 | (46.2%) | 1.44 | 0.40 - 5.23 |  |
| 30% + | 10/14 | (71.4%) | 3.63 | 0.94 - 14.04 |  |
| **Age at baseline** |  |  | 1.02 | 0.84 - 1.24 | 0.839 |
| **Sex** |  |  |  |  |  |
| Male | 14/38 | (36.8%) |  |  |  |
| Female | 41/71 | (57.8%) | 2.34 | 1.04 - 5.27 | 0.039 |
